# Differences in Viral RNA Synthesis but Not Budding or Entry Contribute to the In Vitro Attenuation of Reston Virus Compared to Ebola Virus

**DOI:** 10.3390/microorganisms8081215

**Published:** 2020-08-11

**Authors:** Bianca S. Bodmer, Josephin Greßler, Marie L. Schmidt, Julia Holzerland, Janine Brandt, Stefanie Braun, Allison Groseth, Thomas Hoenen

**Affiliations:** 1 Institute of Molecular Virology and Cell Biology, Friedrich-Loeffler-Institut, 17493 Greifswald-Insel Riems, Germany; bianca.bodmer@fli.de (B.S.B.); Josephin.Gressler@fli.de (J.G.); marie.schmidt@charite.de (M.L.S.); Janine.Brandt@fli.de (J.B.); braunstefanie.erfurt@gmail.com (S.B.); 2 Junior Research Group Arenavirus Biology, Friedrich-Loeffler-Institut, 17493 Greifswald-Insel Riems, Germany; Julia.Holzerland@fli.de (J.H.); allison.groseth@fli.de (A.G.)

**Keywords:** Ebola virus, Reston virus, filovirus, pathogenicity, RNA synthesis, budding, entry, trVLP system

## Abstract

Most filoviruses cause severe disease in humans. For example, Ebola virus (EBOV) is responsible for the two most extensive outbreaks of filovirus disease to date, with case fatality rates of 66% and 40%, respectively. In contrast, Reston virus (RESTV) is apparently apathogenic in humans, and while transmission of RESTV from domestic pigs to people results in seroconversion, no signs of disease have been reported in such cases. The determinants leading to these differences in pathogenicity are not well understood, but such information is needed in order to better evaluate the risks posed by the repeated spillover of RESTV into the human population and to perform risk assessments for newly emerging filoviruses with unknown pathogenic potential. Interestingly, RESTV and EBOV already show marked differences in their growth in vitro, with RESTV growing slower and reaching lower end titers. In order to understand the basis for this in vitro attenuation of RESTV, we used various life cycle modeling systems mimicking different aspects of the virus life cycle. Our results showed that viral RNA synthesis was markedly slower when using the ribonucleoprotein (RNP) components from RESTV, rather than those for EBOV. In contrast, the kinetics of budding and entry were indistinguishable between these two viruses. These data contribute to our understanding of the molecular basis for filovirus pathogenicity by showing that it is primarily differences in the robustness of RNA synthesis by the viral RNP complex that are responsible for the impaired growth of RESTV in tissue culture.

## 1. Introduction

Ebolaviruses comprise a genus in the family of filoviruses (order *Mononegavirales*). They contain a negative-sense, single-strand RNA genome encoding seven structural proteins. Among these, four are ribonucleoprotein complex (RNP) proteins, including the nucleoprotein NP, which encapsidates the viral genome, the transcriptional activator VP30, the polymerase cofactor and interferon (IFN) antagonist VP35, and the viral polymerase L. These RNP proteins are necessary and sufficient for viral RNA synthesis (i.e., replication and transcription of the viral genome) [1]. The remaining viral structural proteins are VP24, which is an interferon antagonist as well as involved in RNP condensation [2,3,4], the matrix protein VP40, which is the driving force for morphogenesis and budding of virions [5], and finally, the glycoprotein GP, which mediates attachment and fusion with target cells (reviewed in [6]).

In many cases, infection with ebolaviruses causes severe hemorrhagic fevers in humans; however, the pathogenic potential of individual virus species is highly variable. The most virulent of the ebolaviruses is Ebola virus (EBOV), which was also one of the first ebolaviruses to be discovered in 1976 [7]. It is responsible for the recent outbreak of Ebola virus disease (EVD) in the Democratic Republic of Congo, with 3463 confirmed cases and 2280 deaths as of June 2020 (case fatality rate of 66%) [8], as well as the West African EVD outbreak in 2013–2016, which was the most devastating ever reported with over 28,600 cases and 11,325 deaths (case fatality rate of 40%) [8,9]. In contrast, Reston virus (RESTV) is apparently apathogenic in humans, leading to seroconversion in the absence of clinical disease (reviewed in [10]). It was first identified in 1989, when cynomolgus monkeys at a primate holding facility in Reston, USA, died of hemorrhagic fever [11,12]. Later, RESTV could also be isolated from domestic pigs co-infected with porcine reproductive and respiratory syndrome virus in the Philippines and China; however, these animals did not show any signs of hemorrhage [13,14]. Differences in the pathogenic potential of EBOV and RESTV have also been described in various animal models, including immune-compromised [15,16] or humanized mouse models [17] and non-human primates [18].

A number of studies have attempted to identify the determinants responsible for this marked difference in pathogenicity, including investigations focused on differences in IFN antagonism by VP24 [19,20] and VP35 [21], as well as contributions to pathogenic potential by GP [22] and differences in host responses to infection with EBOV and RESTV [23]. In particular, an interesting observation reported in several studies is that RESTV is already impaired in its in vitro growth compared to EBOV, even in IFN-deficient cells [22,24], which correlates with marked differences in virus replication seen in vivo, as well as pathogenic potential [17,22,25]. Nevertheless, a full explanation of the molecular basis for the differences in pathogenicity between RESTV and EBOV is still lacking, although this would greatly enhance our ability to evaluate the threat posed by spillover events of RESTV into the human population. Furthermore, an improved understanding of pathogenicity determinants would provide a basis for evidence-based risk assessment for the pathogenic potential of novel filoviruses, such as Lloviu virus (LLOV), the first filovirus found in Europe [26], Měnglà virus, recently identified in China [27], and Bombali virus, a novel ebolavirus discovered in West Africa [28].

While all ebolaviruses are considered biosafety level 4 (BSL-4) agents, life cycle modeling systems allow an analysis of their life cycle without the necessity for a BSL-4 facility (reviewed in [29]). Maybe even more importantly, they allow a reductionist approach analyzing individual aspects of the viral life cycle and provide a system in which different virus components can easily be exchanged. These systems are based on minigenomes, i.e., miniature versions of the viral genome in which viral genes have been removed and replaced with a reporter gene, while the non-coding terminal regions (called leader and trailer) that carry sequence signals important for recognition by the viral RNP proteins remain unchanged. Expression of such monocistronic minigenomes in mammalian cells together with the RNP proteins results in minigenome replication and transcription (i.e., viral RNA synthesis), and subsequent translation of minigenome-encoded reporter mRNAs leads to reporter activity, reflecting these processes. By also including the viral genes for VP40, VP24, and GP (either from expression plasmids or the minigenome itself, i.e., as a tetracistronic minigenome), transcription and replication-competent virus-like particles (trVLPs) can be formed, which incorporate minigenomes and can infect target cells, where these minigenomes can again be replicated and transcribed by RNP proteins expressed in these target cells from plasmids. The resulting reporter activity in target cells in such a tetracistronic trVLP system is thus dependent on viral RNA synthesis and protein production as well as morphogenesis, budding, and entry.

Here, we used life cycle modeling systems to systematically assess and compare the kinetics of budding, entry, and RNA synthesis of RESTV and EBOV in isolation. Our data suggest that it is reduced efficiency of the RESTV RNP proteins in mediating viral RNA synthesis, rather than differences in entry or budding, that is responsible for the impaired growth of RESTV in vitro, and thus contributes to the differences in pathogenicity between these viruses.

## 2. Materials and Methods

### 2.1. Cells

293T (human embryonic kidney cells, Collection of Cell Lines in Veterinary Medicine CCLV-RIE 1018) and Vero E6 (African green monkey kidney cells, kindly provided by Stephan Becker, Philipps University Marburg) cells were cultured in Dulbecco’s modified Eagle’s medium (DMEM; ThermoFisher Scientific, Darmstadt, Germany) supplemented with 10% fetal calf serum (FCS), 100 U/mL penicillin, 100 µg/mL streptomycin (PS; ThermoFisher Scientific), and 1× GlutaMAX (ThermoFisher Scientific).

For the differentiation of primary human macrophages, whole blood from healthy anonymous donors obtained from the blood bank of the Greifswald University Hospital was used. Peripheral blood mononuclear cell (PBMC) isolation was performed, as described in [30]. Briefly, blood was mixed 1:1 with PBS, layered onto 1.077 g/mL Ficoll-Paque Plus (GE Healthcare, München, Germany, and centrifuged for 45 min at 500× *g* without brake. The PBMC layer at the serum-Ficoll-Paque Plus interface was isolated, and PBMCs were washed twice with PBS supplemented with 1% FCS and centrifuged at 200× *g* for 10 min. Erythrocytes were removed by incubation with erythrocyte lysis buffer (0.15 M NH_4_Cl, 10 mM KHCO_3_, 0.1 mM EDTA) for 5 min. Afterward, PBMCs were washed as before and resuspended in RPMI-1640 supplemented with 2% human AB serum (Sigma, München, Germany. For freezing, cells were mixed 1:1 with freezing medium (80% FCS, 20% DMSO (Carl Roth, Karlsruhe, Germany)).

For infection experiments, PBMCs were thawed in RPMI-1640 supplemented with PS and 10% FCS and centrifuged for 10 min at 200× *g*, followed by two washing steps with 10 mL warm PBS with 1% FCS and centrifugation as before. PBMCs were seeded in Primaria 6-well plates (Corning, NY, USA) at a density of 6 × 10^6^ cells in 2 mL of RPMI-1640 supplemented with PS and 5% human AB serum per well. PBMCs were incubated for 1 h to let monocytes attach to the well’s surface. Remaining PBMCs were removed by washing two times with 1 mL PBS supplemented with 1% FCS and then differentiated for 7 days in 2 mL RPMI-1640 supplemented with PS and 5% human AB serum per well.

All cells were incubated at 37 °C with 5% CO_2_.

### 2.2. Plasmids

Components for the mono- and tetracistronic minigenome assay, including expression plasmids encoding the EBOV or RESTV RNP proteins, T7 polymerase, firefly luciferase, T-cell immunoglobulin and mucin domain 1 (TIM-1) and the EBOV mono- and tetracistronic minigenomes (p1cis-EBOV-vRNA-hrLuc, p4cis-EBOV-vRNA-hrLuc) have been previously described [4,22]. Heterologous exchange of the VP40 and GP genes in the tetracistronic minigenome was done using a type IIS restriction enzyme-based approach, removing the VP40 and GP open reading frames from p4cis-EBOV-vRNA-hrLuc but leaving the start and stop codon and two BsmBI and one XhoI restriction sites, which are not part of the original gene. The RESTV monocistronic minigenome was generated by exchanging the chloramphenicol acetyltransferase (CAT) reporter from a previously described T7-driven minigenome [31] against hrLuc using a type IIS restriction enzyme-based approach. Detailed cloning strategies are available upon request.

### 2.3. Viruses

Zaire ebolavirus rec/COD/1976/Mayinga-rgEBOV (GenBank accession number KF827427.1), which is identical in sequence to the EBOV Mayinga isolate except for four silent mutations used as genetic markers [32], and Reston ebolavirus rec/USA/1989/Philippines89-Pennsylvania-rgRESTV, which is identical in sequence to the RESTV Pennsylvania isolate except for five silent mutations used as genetic markers (c814g; g7252a; t8582c; t8996a; c17546t), were used for infection experiments. EBOV and RESTV were propagated in VeroE6 cells, and virus titers were determined by 50% tissue culture infectious dose (TCID_50_) assay. All work with infectious virus was performed under BSL-4 conditions at the Friedrich-Loeffler-Institut (Federal Research Institute of Animal Health, Greifswald Insel-Riems, Germany) following approved standard operating procedures.

### 2.4. Growth Kinetics and Virus Titration

Vero E6 cells or primary human macrophages (about 90% confluence) were infected with 1 × 10^4^ TCID_50_ RESTV or EBOV (MOI = 0.01) on day 0 for 1 h. After washing three times with the respective serum-free medium, 4 mL culture medium (with added supplements and serum or FCS, see above) was added to the cells. From day 0 to 7 post-infection, 500 µL of supernatant was harvested and stored at −80 °C until titration, and 500 µL fresh culture medium was added to replace the volume collected. Virus titers in supernatants from days 0 to 7 post-infection were determined by TCID_50_ assay in Vero E6 cells [33]. The lower limit of detection was 6.3 × 10^1^ TCID_50_.

### 2.5. Minigenome and trVLP Assays

For monocistronic minigenome assays, 293T cells were transfected with plasmids encoding a codon-optimized T7-polymerase (125 ng), firefly luciferase (as a control, 125 ng), the T7-driven monocistronic minigenome (pT7-EBOV-1cis-vRNA-hrLuc; 125 ng or pT7-RESTV-1cis-vRNA-hrLuc; 125 ng), and pCAGGS-based expression plasmids for the EBOV or RESTV RNP proteins NP (62.5 ng), VP35 (62.5 ng), VP30 (37.5 ng), and L (500 ng), unless otherwise indicated. In titration experiments, the amount of plasmid DNA in all samples was kept constant by adding empty pCAGGS plasmid as necessary. Forty-eight hours after transfection, cells were lysed for 10 min in 200 µL 1× Lysis Juice (PJK, Kleinblittersdorf, Germany), and the cell debris was removed by centrifugation for 3 min at 10,000× *g*. Then 40 µL of lysate was mixed with 40 µL Renilla-Glow Juice (PJK) or 40 µL Beetle Juice (PJK) in opaque 96-well plates, and luminescence was measured using a Glomax Multi (Promega, Walldorf, Germany) microplate reader.

Tetracistronic minigenome assays were performed as previously described [4], with the indicated modifications to assess individual steps of the virus life cycle. 293T cells were transfected with plasmids encoding a codon-optimized T7-polymerase (125 ng), a tetracistronic minigenome (125 ng), and expression plasmids for the EBOV RNP proteins NP (62.5 ng), VP35 (62.5 ng), VP30 (37.5 ng), and L (500 ng). trVLPs in the supernatant were harvested at the time points indicated below for the different experiments and used to infect 293T cells pre-transfected with expression plasmids for TIM-1 and EBOV or RESTV RNP proteins. Non-optimized plasmid amounts in these target cells were NP: 62.5 ng, VP35: 62.5 ng, VP30: 37.5 ng, and L: 500 ng, for both RESTV and EBOV, whereas optimized plasmid amounts were NP: 210.9 ng, VP35: 27.8 ng, VP30: 84.4 ng, and L: 500 ng for RESTV and NP: 140.6 ng, VP35: 93.8 ng, VP30: 56.3 ng, and L: 500 ng for EBOV. Transfected target cells were lysed, and the reporter activity was measured at the indicated time points post-infection with trVLPs.

For the assessment of entry, EBOV tetracistronic minigenomes encoding either EBOV GP, RESTV GP, or no GP were used. trVLPs were harvested 48 h post-transfection and used to infect target cells pre-transfected with non-optimized amounts of EBOV RNP protein plasmids. Reporter activity was then measured at the time points indicated in the figures. The efficiency of morphogenesis and budding was compared using EBOV tetracistronic minigenomes encoding either EBOV VP40, RESTV VP40, or no VP40. trVLPs were harvested at time points indicated in the figures and used to infect target cells pre-transfected with expression plasmids for TIM-1 and EBOV RNP proteins, as described above. The reporter activity was measured 48 h post-infection. For the assessment of the efficiency of RNA synthesis, EBOV trVLPs harvested 48 h post-transfection were used to infect target cells pre-transfected with expression plasmids for EBOV or RESTV RNP proteins (also omitting the plasmid encoding L as a negative control), as indicated. The reporter activity was measured at the time points post-infection indicated in the figures.

### 2.6. Statistical Analysis

**Figure 1:**
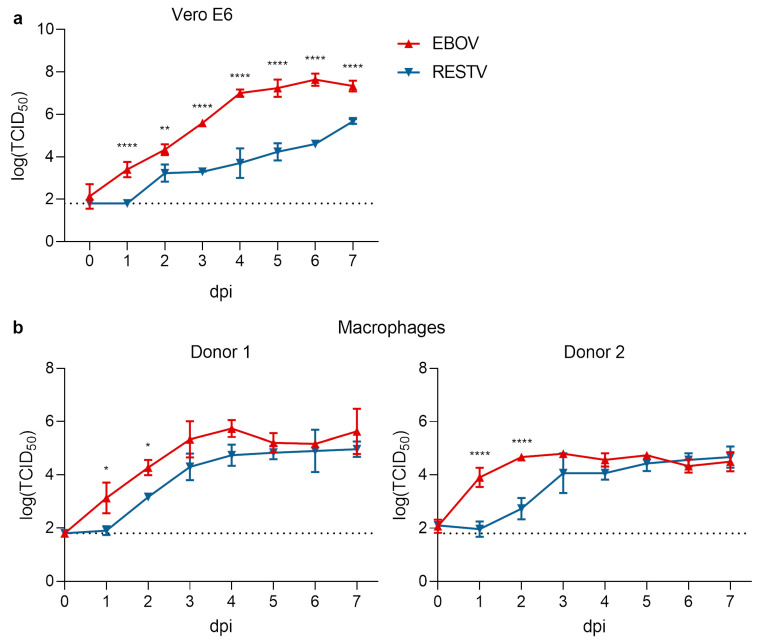
Comparison of in vitro growth kinetics of Ebola virus (EBOV) and Reston virus (RESTV). (**a**) Growth kinetics in Vero E6 cells infected with 1 × 10^4^ 50% tissue culture infectious dose (TCID_50_) (multiplicity of infection, MOI = 0.01) of EBOV (red) or RESTV (blue). (**b**) Growth kinetics in primary monocyte-derived human macrophages infected with 1 × 10^4^ TCID_50_ (MOI = 0.01) of EBOV (red) and RESTV (blue). Viral titers (TCID_50_) in the supernatant at the indicated days post-infection (dpi) are shown. The dotted line indicates the limit of detection of the TCID_50_ assay. In all panels, means and standard deviations of 3 biological replicates are shown. *: *p* < 0.5; **: *p* < 0.01; ****: *p* < 0.0001.

**Figure 2:**
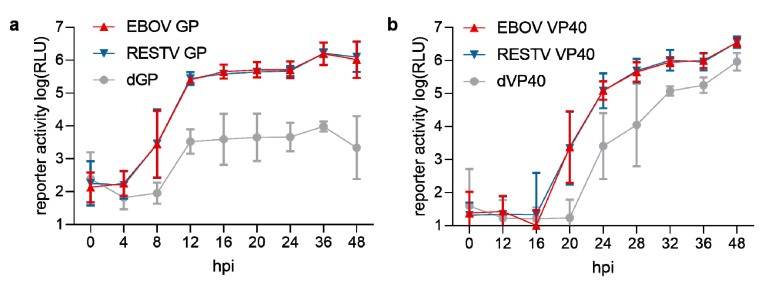
Comparison of EBOV and RESTV entry and budding efficiencies. (**a**) Kinetics of entry for trVLPs carrying either EBOV GP (red), RESTV GP (blue), or no GP (dGP, grey) on their surface. trVLPs were produced in 293T cells by transfection of all necessary plasmids, including a tetracistronic minigenome encoding the different GP variants, harvested, and used to infect 293T target cells pre-transfected with expression plasmids for EBOV RNP proteins. The target cells were lysed at the depicted time points post-infection, and the luciferase activity was measured. (**b**) Budding efficiencies of trVLPs produced by EBOV VP40 (red), RESTV VP40 (blue), or no VP40 (dVP40, grey). trVLPs were produced, as described above, but using tetracistronic minigenomes encoding either EBOV VP40, RESTV VP40, or no VP40. trVLPs were harvested at the depicted time points post-transfection and used to infect 293T target cells pre-transfected with expression plasmids for EBOV RNP proteins. Means and standard deviations of 3 (**a**) or 6 (**b**) independent experiments are shown. trVLPs, transcription and replication-competent virus-like particles; GP, glycoprotein; RNP, ribonucleoprotein.

**Figure 3:**
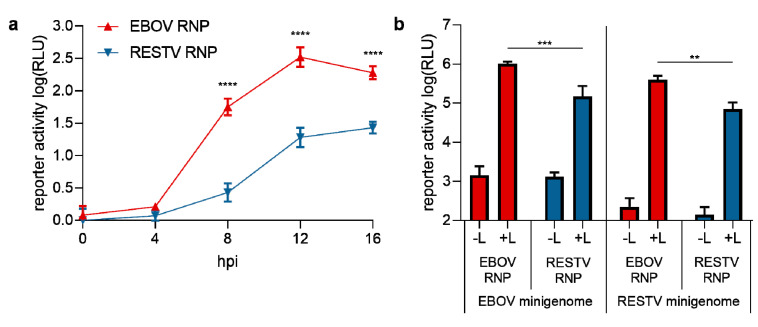
Comparison of the efficiency of EBOV and RESTV RNA synthesis. (**a**) Kinetics of viral RNA synthesis following EBOV trVLP infection by EBOV (red) or RESTV (blue) RNP proteins. trVLPs were produced by transfecting 293T cells with an EBOV tetracistronic minigenome plasmid and expression plasmids for EBOV RNP proteins and T7 polymerase. trVLPs were harvested and used to infect 293T target cells pre-transfected with expression plasmids for either EBOV or RESTV RNP proteins. At the depicted time points, target cells were lysed, and the luciferase activity was measured. For the determination of background, the expression plasmid for L was replaced by an empty vector, and these background values were subtracted from the data. (**b**) Kinetics of viral RNA synthesis from EBOV or RESTV monocistronic minigenomes by EBOV (red) or RESTV (blue) RNP proteins. 293T cells were transfected with expression plasmids for a monocistronic minigenome for EBOV or RESTV, EBOV or RESTV RNP proteins, as indicated. Cells were lysed 48 h post-transfection, and the luciferase activity was measured. Means and standard deviations of 3 biological replicates from 2 independent experiments (**a**) or 3 independent experiments (**b**) are shown. **: *p* < 0.01; ***: *p* < 0.001; ****: *p* < 0.0001.

Two-way ANOVAs with Tukey’s (Figures 2 and 3b) or Sidak’s (Figures 1, 3a and 5) multiple comparisons tests were performed using GraphPad Prism 8.1.0. (San Diego, CA, USA).

## 3. Results

### 3.1. RESTV Growth Is Impaired Compared to EBOV in Both IFN-Deficient and Competent Cells

In previous studies, RESTV has been reported to grow slower than EBOV in IFN-deficient Vero cells [22,24]. As a starting point for our studies, we, therefore, performed growth kinetics for RESTV or EBOV in Vero E6 cells to confirm these observations (Figure 1a). Indeed, we also observed that RESTV grew significantly slower and to lower titers than EBOV in this cell type. In order to expand these studies to a cell type that is more relevant, we chose macrophages as one of the first and most prominently infected cell types during filoviruses infection in vivo [34]. To this end, we compared the growth kinetics of RESTV and EBOV in primary human macrophages isolated from two different donors (Figure 1b). While at 3 days post-infection the titers of RESTV and EBOV in infected macrophages were comparable and had reached a plateau, for the first two days after infection RESTV growth was delayed, showing significant differences in titer of between 13- and 100-fold.

### 3.2. EBOV and RESTV GP and VP40 Have Identical Entry and Budding Efficiency

The observed growth impairment of RESTV already in vitro, and particularly in IFN-deficient cell types, indicates that there must be fundamental differences in the efficiency of basic aspects of the virus life cycle between EBOV and RESTV. To identify which step in the viral life cycle was impaired, we decided to use the tetracistronic trVLP system, as this allows us to easily make specific genetic modifications aimed at dissecting individual aspects of the virus life cycle. In particular, we analyzed the impact of exchanging the RESTV and EBOV proteins responsible for entry (i.e., GP), budding (i.e., VP40), or replication and transcription (i.e., the RNP proteins) in the trVLP system.

In order to take a closer look at entry kinetics, we exchanged the EBOV GP gene in the tetracistronic minigenome plasmid with the RESTV GP gene or deleted it completely as a control for non-specific trVLP uptake. We then determined entry kinetics of the resulting EBOV GP- and RESTV GP-bearing trVLPs by measuring reporter activity in target cells at different time points after infection. Infection with both types of trVLPs resulted in virtually identical reporter activities at all time points after infection, and also the maximum levels of reporter activity were indistinguishable (Figure 2a).

Similarly, for the analysis of budding kinetics, the VP40 gene of EBOV was exchanged against the RESTV VP40 gene (or deleted as a control for residual VP40-independent budding, most likely mediated by GP [5]), and the trVLPs produced were harvested at different time points post-transfection and subsequently used for the infection of target cells (Figure 2b). Again, no differences in reporter activity, indicating the amount of produced trVLPs present, were observed between trVLP preparations produced by either EBOV or RESTV VP40 up to 48 h post-transfection.

### 3.3. EBOV and RESTV RNP Proteins Show Different Efficiencies in Viral RNA Synthesis

Since the entry of trVLPs containing heterologous GP and budding of trVLPs containing heterologous VP40 did not show any differences between RESTV and EBOV, this suggested possible differences in viral RNA synthesis as the remaining key aspect of the viral life cycle. To investigate this possibility, we used trVLPs to infect target cells that had been pre-transfected with expression plasmids for either EBOV or RESTV RNP proteins. At 8 h post-infection, cells expressing the RESTV RNP proteins showed 21-fold less reporter activity compared to cells expressing the EBOV RNP proteins, and also at 12 and 16 h post-infection differences remained significant (17- and 7-fold, respectively) (Figure 3a). This suggests that RESTV RNP proteins show a reduced efficiency in their ability to mediate viral RNA synthesis compared to EBOV RNP proteins.

However, these observed differences in RNP efficiency in viral RNA synthesis could also be a result of compatibility issues between the RESTV RNP proteins and the EBOV minigenome, which contains EBOV leader and trailer regions. To exclude this possibility, we further compared the activity of EBOV and RESTV RNP proteins on both EBOV and RESTV minigenomes. Results showed 6 to 7-fold reduced reporter activity, reflecting viral RNA synthesis, in classical minigenome assays determined 48 h post-transfection for RESTV RNP proteins independent of the origin of the minigenome (Figure 3b). This indicates that it is the origin of the RNP proteins and not the origin of the minigenome or compatibility issues that was responsible for the observed differences in the efficiency of RNA synthesis.

### 3.4. Even under Optimized Conditions, RESTV RNP Proteins Are Less Efficient in Viral RNA Synthesis Than EBOV RNP Proteins

**Figure 4:**
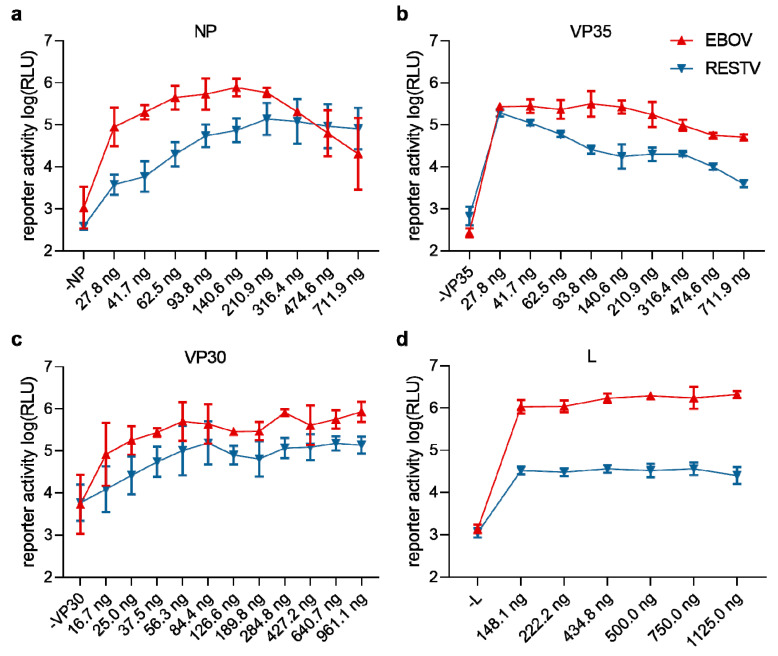
Optimization of RESTV and EBOV RNP expression plasmid concentrations. Reporter activity, reflecting viral RNA synthesis, in 293T cells transfected with an EBOV monocistronic minigenome plasmid and expression plasmids for RNP proteins for either RESTV (blue) or EBOV (red) is shown. Plasmid amounts for NP (**a**), VP35 (**b**), VP30 (**c**), or L (**d**) were titrated, as indicated. As a negative control, each of the respective plasmids was replaced by an empty vector. Means and standard deviations of 3 independent experiments are shown.

For our initial comparison of RNP efficiencies, we kept the amounts of transfected plasmids identical between EBOV and RESTV, using the amounts that had been previously determined to be optimal for the EBOV minigenome system [35]. To exclude that the observed difference in efficiency between EBOV and RESTV RNPs was due to non-optimal amounts of the transfected RESTV RNP plasmids, we titrated and optimized the amounts of RNP protein-encoding plasmids for both EBOV and RESTV in the context of an EBOV minigenome (Figure 4). While in the case of NP, VP30, and VP35 changing the amounts of transfected plasmid clearly influenced the levels of viral RNA synthesis (Figure 4b,c), in almost all cases we observed that RESTV RNP proteins resulted in less RNA synthesis than their EBOV counterparts. Only in the case of NP, at high levels of transfected plasmids did RNA synthesis driven by RESTV RNP proteins exceed that driven by EBOV RNP proteins; however, in these cases, the amounts of EBOV NP were clearly suboptimal (Figure 4a).

**Figure 5:**
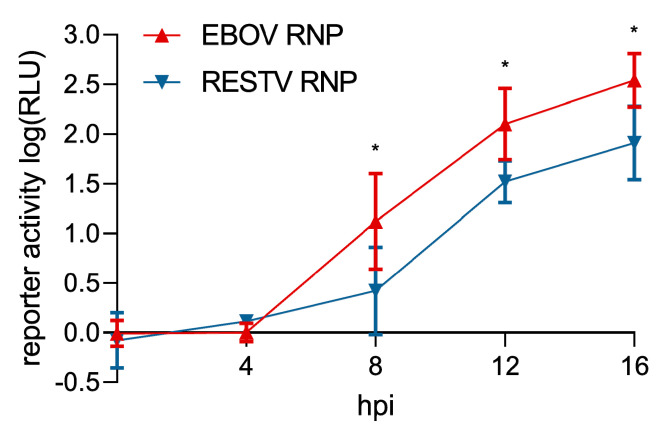
Comparison of efficiencies of EBOV and RESTV RNA synthesis using optimized RNP concentrations. Cells were pre-transfected with optimized amounts of either EBOV (red) or RESTV (blue) RNP protein-expressing plasmids, as determined in Figure 4, and infected with EBOV trVLPs. At the depicted time points, target cells were lysed, and the luciferase activity was measured. As negative controls, the expression plasmid for EBOV L was replaced by an empty vector, and the background values were subtracted from the data. Means and standard deviations of 4 independent experiments are shown. *: *p* < 0.5.

These titration experiments now allowed us to repeat the trVLP experiment comparing the efficiency of EBOV and RESTV RNP proteins using optimized amounts of RNP plasmids for each virus. While differences were less pronounced under optimized conditions, we still observed that RNA synthesis driven by RESTV RNP proteins was less efficient compared to EBOV RNP proteins, with differences visible starting at 8 h post-infection (Figure 5). This further supports that an intrinsic difference in the efficiency of the RNP proteins driving viral RNA synthesis, but not differences in the efficiency of budding or entry, is responsible for the impaired growth of RESTV in vitro.

## 4. Discussion

A number of studies suggest that differences in the pathogenic potential of EBOV and RESTV are most likely not caused by one specific determinant, but rather are the result of multiple factors [19,20,21,22,23]. However, it is striking that already in vitro, and even in IFN-deficient cells, RESTV shows a clear growth impairment, indicating that there must be differences in the efficiency of very basic aspects of the virus life cycle. In this study we have shown that these differences are not at the level of budding or entry, but rather at the level of RNA synthesis.

This finding is consistent with previous reports using minigenome systems featuring CAT as a reporter, which have also suggested that there may be differences in the efficiency of viral RNA synthesis between RESTV and EBOV [24,31]. However, our results also clearly show that it is the origin of the RNP proteins, rather than regulatory RNA elements in the genome termini, or their compatibility with the RNP proteins, that is responsible for this difference. Interestingly, EBOV RNP proteins also show a higher efficiency of viral RNA synthesis in comparison to other newly discovered filoviruses with potentially restricted pathogenicity, for example, when comparing them with RNP proteins from the related LLOV [36]. Again, this is seen irrespective of whether the RNPs are acting on an EBOV minigenome or a LLOV minigenome. The evolutionary reasons for these differences in the efficiency of RNA synthesis between different filovirus species remain enigmatic, and their elucidation will require identification of the natural hosts of these viruses, followed by careful studies of virus-host interactions in these hosts.

Of course, when interpreting the results from these experiments one important consideration is the expression levels of EBOV or RESTV RNP proteins, particularly since our study, as well as previous studies, have shown that the ratios of RNP proteins are crucial for efficient RNA synthesis [1,24,31,35]. In this context, the key question is what constitutes the most appropriate expression level of EBOV/RESTV RNP proteins for a fair comparison of the efficiency of these proteins. One approach would be to use identical amounts of DNA plasmids for EBOV/RESTV proteins or to titrate these amounts to obtain the same expression levels for EBOV/RESTV proteins. However, it is not clear whether using expression levels (and thus also protein ratios) that have been optimized for an EBOV minigenome system would be appropriate amounts in a RESTV minigenome system, and whether these levels would correspond to the situation found during infection. To further complicate things, during an infection the expression levels of viral proteins are dependent on the efficiency of RNA synthesis, so that even emulating these levels might not allow an unbiased comparison. Thus, it appears that the most unbiased approach is to determine the optimal expression levels of RNP proteins, independently for both EBOV and RESTV, and compare the efficiencies of RNA synthesis under these conditions. The rationale then is that if even in this optimal situation we still observe a higher efficiency of EBOV RNP proteins vs. RESTV RNP proteins, this clearly suggests that this is also going to be the case under real-life conditions.

A second point that needs to be considered is whether the differences in reporter activity we observe in the trVLP and minigenome assays shown in Figure 3 are due to differences in RNA synthesis or could be attributed to other aspects in the virus life cycle modeled in these assays. In particular, in the case of the trVLP assay entry, uncoating, viral genome replication, viral mRNA transcription, and finally mRNA transport to ribosomes and translation of these mRNAs all have to occur in order to result in reporter protein expression and reporter activity. However, since in this assay the incoming trVLPs are identical (regardless of whether EBOV or RESTV RNP proteins are transfected into the target cells), one can reasonably assume that entry and uncoating will occur with the same efficiency. Further, the mRNAs that are produced after genome replication and transcription in either assay are also identical, so that it is unlikely that there are differences in their transport, stability, or translation efficiency. Thus, RNA synthesis (i.e., genome replication and transcription) remains as the only step of the virus life cycle where differences in the origin of the RNP proteins could result in differences in reporter activity, which is exactly what we observe.

Finally, with respect to the compatibility of RNP proteins and minigenomes, the experiments shown in Figure 3b were only done in the context of monocistronic minigenome assays, but not in the context of a tetracistronic trVLP assay, since to the best of our knowledge a tetracistronic trVLP system for RESTV currently does not exist. This raises questions about the extent to which results from the monocistronic minigenome also apply in the experimental context of a tetracistronic trVLP assay. In this regard, it is important to note that the promoter elements for replication, as well as transcription (particularly of the reporter gene), are present in the (mini-)genome termini [37,38,39], which are identical between mono- and tetracistronic minigenomes. Further, our results clearly show that in the context of a monocistronic minigenome it is not the phylogenetic relationship of the minigenome and the RNP proteins (and thus interspecies compatibility between these regulatory RNA elements and the RNP proteins), but rather the origin of the RNP proteins that is responsible for differences in the efficiency of RNA synthesis. Together, this then clearly suggests that, also in the context of a tetracistronic trVLP assay, interspecies compatibility between regulatory RNA elements and RNP proteins should not play a role. However, we cannot exclude that differences in interactions of VP40 and/or VP24 (which are expressed from the tetracistronic minigenome) with RNP proteins might play a role since both these proteins have been shown to modulate RNA synthesis [37,40]. It is conceivable that the strength of these interactions might be dependent on the phylogenetic relationship of the proteins involved. However, given that VP40 and VP24 are only expressed at later points during the experiment (as demonstrated by the fact that VP40-mediated budding occurs only after 20 h), whereas we observe differences in the efficiency of RNA synthesis early in the experiment (starting at 8 h), such factors appear unlikely to contribute to the observed differences.

Previous studies have so far not examined the contributions of differences in other aspects of the viral life cycle (i.e., morphogenesis/budding and entry) to robust filovirus growth. In this respect, our results clearly show that fundamental differences in morphogenesis/budding and entry, mediated by the viral proteins GP and VP40, do not contribute to the observed differences in the growth kinetics of EBOV and RESTV in vitro. Nonetheless, in vivo these proteins could still contribute to differences in pathogenic potential between these two viruses, and also tissue-specific differences in infection efficiency could be contributing factors in an organism setting. For example, it has been shown in the IFN^-/-^ mouse model that recombinant wild-type EBOV infects both Kupffer cells and hepatocytes, whereas a recombinant chimeric EBOV expressing RESTV-GP is mainly restricted to Kupffer cells, resulting in marked differences in necrosis and inflammation in the liver [22]. This is supported by data from humanized mice, where lethal infection with either EBOV or RESTV was associated with high levels of inflammation, replication, and cell death in the liver, whereas this was not the case for mice surviving RESTV infection [17,25]. It will thus be interesting in the future to expand our studies of RESTV attenuation mechanisms to different primary cells from the liver and other organs, in order to better understand the contributions of entry, budding, and viral RNA synthesis to the observed differences in infection in these organs and tissues.

In summary, our study clarifies the mechanistic basis for the growth impairment observed for RESTV infection in tissue culture, indicating that differences in the efficiency of viral RNA synthesis but not differences in entry and budding are responsible for this phenomenon, and contributes to an overall understanding of pathogenicity factors of filoviruses, which are clearly multifactorial in nature.
